# Current review of acute type A aortic dissection in Malaysia

**DOI:** 10.1007/s12055-023-01608-2

**Published:** 2023-11-14

**Authors:** Paneer Selvam Krishna Moorthy, Abdul Samad Sakijan, Deventhiran Permal, Intan Fariza Gaaffar, Aini Syakirin Kepli, Haidatul Insyirah Sahimi

**Affiliations:** 1 Department of Cardiothoracic & Vascular Surgery, National Heart Institute, 145 Jalan Tun Razak, 50400 Kuala Lumpur, Malaysia; 2 Department of Imaging & Non-Invasive Laboratory, National Heart Institute, 145 Jalan Tun Razak, 50400 Kuala Lumpur, Malaysia; 3 Clinical Research Department, National Heart Institute, 145 Jalan Tun Razak, 50400 Kuala Lumpur, Malaysia

**Keywords:** Aortic dissection, Type A, Review, Diagnosis, Treatment

## Abstract

Acute type A aortic dissection (ATAAD) still poses significant challenges and management dilemmas for cardiovascular surgeons worldwide. Despite the continuous improvement in diagnosis and management strategies for ATAAD, clinical outcomes remain poor and the optimal therapy is still debatable especially those with malperfusion syndrome (MPS). This review is based on the current literature and includes the results from the Aortic Registry of National Heart Institute of Malaysia (NHIM) database. It covers different aspects of ATAAD and concentrates on the outcome of surgical repair. The diagnosis is often delayed leading to variable outcomes. High index of suspicion and urgent treatment is required to tackle this dynamic disease which include the variation in presentation and clinical course. Different surgical techniques and perfusion strategies have been described to save patients. Complex techniques such as total arch replacement (TAR) with frozen elephant trunk and valve sparing root surgery may provide long-term benefit in selected patients, but require significant surgical expertise and experience.

## Introduction

True aortic dissection (AD) is defined as separation of the medial layer of the aortic wall leading to formation of a true lumen (TL) and false lumen (FL) with or without communication. It is a life-threatening condition of the aorta, and associated with significant morbidity and mortality [1]. The AD involving the ascending aorta is commonly referred to as acute type A aortic dissection (ATAAD) carries a grave prognosis without immediate surgical repair. The patients usually die from complications which, includes rupture of the aorta, pericardial tamponade, acute aortic regurgitation or acute heart failure, and malperfusion syndrome (MPS) [2–5].

Many countries and regions have established multicenter registration studies [4–10]. The International Registry of Aortic Dissection (IRAD) established in 1996 has published several studies that reported a significant impact on the diagnosis and treatment of aortic dissection worldwide [9, 10]. In 2018, the Registry of Aortic Surgery (RAS) was established at National Heart Institute of Malaysia (NHIM), Malaysia, in accordance with the model of the IRAD. The aim of this review was to report actual preoperative management, type and extent of surgery, and outcomes of surgical treatment for aortic disease in NHIM, Malaysia.

An analysis performed on 413 patients presented with ATAAD to NHIM between January 1997 and July 2022. In this review, we mainly focus on articles on epidemiology, classification, risk factors, clinical presentation, diagnostic imaging, treatment, complications, and long-term follow-up of ATAAD in the past 20 years.

### Definitions and classification systems

AD is now defined as part of clinical spectrum of acute aortic syndromes (AAS) [11–13]. AAS are defined as emergency conditions with the breakdown of the intima and media resulting in intramural hematoma (IMH), penetrating aortic ulcers (PAU), frank AD, and aortic rupture [13]. Traumatic dissections occur from tears of the intima secondary to fall, motor vehicle accident, and iatrogenic injuries, such as arterial cannulation for cardiopulmonary bypass (CPB), during percutaneous coronary intervention (PCI), cardiac catheterization, endovascular aneurysm repair (EVAR), transcatheter aortic valve implantation (TAVI), or insertion of an intra-aortic balloon-pump (IABP) [12, 13].

AD is classified based on anatomical location of the aorta tear and chronicity of onset of symptom. A new time classification of AD has been proposed by the IRAD [10]. When survival curves constructed, 4 distinct time periods were noted: hyperacute (symptoms onset to 24 h), acute (2–7 days), subacute (8–30 days), and chronic (> 30 days). The overall survival rate was progressively lower through the 4 time periods [10].

The anatomical classification of Stanford system was introduced in 1970 [14, 15]. Stanford type A AD involve the ascending aorta proximal to the innominate artery (brachiocephalic trunk), regardless of extension into the aortic arch or further down. Stanford type B dissections involve only the thoracic aorta below the left subclavian artery [3, 14, 15]. The Stanford classification is not a classification of the tear site. Consequently, an intimal tear distal to the left subclavian artery with retrograde dissection into the ascending aorta will be classified as being type A. By analogy, intimal tear and dissection originating in the aortic arch will be classified as being type A if extending into the ascending aorta, and as type B if extending into the descending aorta, and as non-A non-B if contained in the arch only [3, 14, 15].

According to the modified DeBakey classification from 1982, both De Bakey type I and type II dissections involve the ascending aorta, but type I extends beyond the innominate artery, while type II is located entirely in the ascending aorta [14]. Thus, Stanford type A dissections include both type I and type II DeBakey dissections and Stanford type B dissections are defined as DeBakey type III dissections. The Stanford classification is useful in guiding acute management, whereas the DeBakey classification is more informative for the purpose of long-term follow-up.

A recent method of categorizing type A dissections by their clinical presentation is the Penn classification [15–17]. Class Aa is characterized by the absence of branch vessel malperfusion or circulatory collapse, and class Ab by symptoms or signs of localized organ ischemia. Class Ac is characterized by circulatory collapse, with or without cardiac involvement, and class Abc is characterized by localized and generalized ischemia combined. The classifications of ATAAD are summarized in Table 1.

**Table 1:**
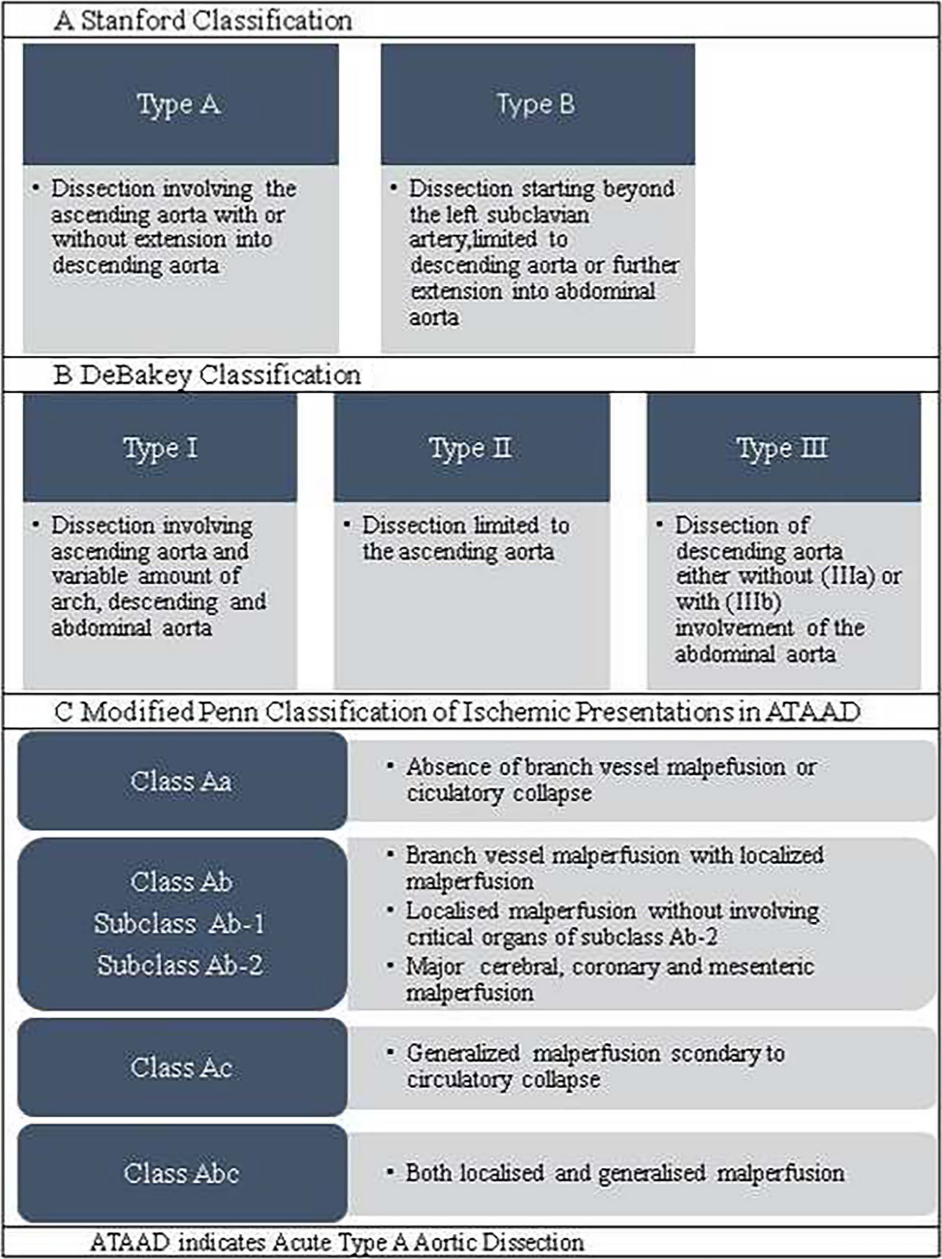
Summary of classifications of ATAAD

### Epidemiology

The incidence of AD is estimated at 6 per 100,000 persons per year [1, 18]. The IRAD series revealed 67% of patients presented with type A AD and remaining 33% type B. Two-third were men and the mean age was 63 years [6–10]. The MPS occurs anywhere from 16 to 34% and can happen with both acute type A and type B dissection [5–10]. It is the second most common lethal complication of ATAAD after rupture.

It is estimated that aortic disease has a prevalence of 15,299 cases among the total adult population in Malaysia of approximately 33.5 million [19]. The annual mortality rate in Malaysia was 5.2 per 100,000 while the Annual Years of Healthy Life Lost was 73.4 per 100,000 in the year 2013 [19, 20].

All races and ethnicity in Malaysia are generally affected by aortic disease [20, 21]. However, there may be differences in terms of susceptibility among the 4 main races in the nation. In a study by Murty et al. done in the state of Sarawak, aortic aneurysms and dissection were more common in the Chinese, contributing 69% of cases, followed by Malay (17%), Indian (7%), and others (7%) [21].

In last 8 years, between 2015 and 2022, it has been documented 462 cases of ATAAD presenting to 22 hospitals in Malaysia, with average 58 cases per year. Out of this, 362 patients (78%) were treated in NHIM. It is obvious that we treat most of the ATAAD in the country.

For the purpose of this review, we have decided to perform a retrospective study on 413 patients who presented to NHIM with ATAAD from 1997 to July 2022. This number is probably an underestimation of overall incidence as many patients die outside hospital or during transfer without diagnosis. These patients are not captured in our census.

### Demographics and risk factors

A retrospective analysis of 413 patients performed and summarized (Table 2). The mean age of all patients was 51.6 ± 12.7 years, and 299 (72.4%) of the patients were male. A history of hypertension 311 was elicited in 72.4% of patients, hyperlipidemia in 35.1%, diabetes in 12.3%, coronary heart disease in 15.7%, and Marfan syndrome was present in 6.1%. Approximately 19.1% of patients had previous open-heart surgery, and the prevalence of cardiac surgery history was 4.2%. Nearly 12.3% of the patients had a history of smoking. About 92(22.3%) patients presented with MPS in ATAAD to National Heart Institute of Malaysia (NHIM). One-third patients with MPS presented with renal MPS: 29(32%) where else others were as follows: coronary MPS: 22 (24%); cerebral MPS: 20 (22%); limb MPS: 12 (13%); mesenteric MPS: 9 (10%); spinal MPS: 3 (3%). The incidence from our series correlates with the IRAD cohort study [9, 10].

**Table 2:** Demographics and history of patients with ATAAD

Category	Total (*n*=413)
Demographics	
Age, mean ± SD Male Female	51.6 ± 12.7 299 (72.4%) 114 (27.6%)
Ethnicity	
Malay Chinese Indian Others	140 (33.9) 233 (56.4) 10 (2.4) 30 (7.3)
Patient history	
Hypertension Hyperlipidemia Diabetes mellitus Smoking Marfan syndrome Previous cardiac surgery Coronary artery disease	311 (75.3%) 145 (35.1%) 51 (12.3%) 51 (12.3%) 25 (6.1%) 79 (19.1%) 65 (15.7%)
Presentation	
Acute Subacute	309 (75%) 104 (25%)
Malperfusion syndrome	
Renal MPS Coronary MPS Cerebral MPS Limb MPS Mesenteric MPS Spinal MPS	29 (32%) 22 (24%) 20 (22%) 12 (13%) 9 (10%) 3 (3%)

Values are in *n* (%) and mean ± standard deviation. ATAAD indicates acute type A aortic dissection; *MPS* malperfusion syndrome

### Clinical presentation

ATAAD is characterized by a sudden onset of intense chest pain and or back ache, often described as “ripping” or “tearing”; this occurred in 85 to 90% of patients in various series including IRAD, German Registry for Acute Aortic Dissection Type A (GERAADA), Nordic Consortium for Acute Type A Aortic Dissection (NORCAAD), and Japanese Registry [4–10]. Migratory pain was observed in almost 15% in IRAD series. In one-third of the patients, the symptoms are accompanied with malperfusion due to impaired flow in end-organ arteries [6, 10]. Lower-extremity malperfusion and pulse deficit are easily detected clinically, while signs of intestinal and renal ischemia may be more difficult to detect in the acute phase. In rare cases, cerebral ischemia or paraplegia are the first presenting symptoms of acute aortic dissection [3]. electrocardiogram (ECG) may show signs of cardiac ischemia or non-specific ST and T-wave alterations, and occasionally coronary flow may be impaired due to coronary ostia dissection [10]. Acute chest pain may a lead to the suspicion of acute coronary syndrome, and as the ECG may indicate ischemia, patients are given anti-thrombotic treatment and transported to coronary intervention units, leading to delay in correct diagnosis and perioperative bleeding complications.

Our series in NHIM also share the similar pattern of having almost 90% patient presenting with chest, back, or abdominal pain and 22.3% with MPS.

### Diagnosis

Early suspicion of ATAAD is mandatory and key for successful treatment as that untreated patients have associated mortality rate of 1 to 2% per hour immediately after symptom onset. Conventional chest X-ray can show a wide mediastinum, which is not specific for diagnosis. Computed tomography (CT) scanning of the aorta is the quickest and most accurate method of confirming the ATAADs [22]. The aim is to identify the intimal tears, visualize the true and the false lumens, and evaluate the extent of the dissection and the involvement of the branching arteries of the aorta. CT also aids in the planning of the surgery and cannulation sites. The sensitivity and specificity of CT are excellent [22], but is questionable in patients with poor renal function or allergy to iodinated dye. Other imaging modalities such as magnetic resonance imaging (MRI) or ultrasonography can be considered instead. MRI is seldom used—as it is too time-consuming and still often unavailable, but it may complement CT angiography in follow-up imaging of the aorta [23]. Transthoracic echocardiography (TTE) reveals the functional state of the heart, valves, and aortic root, and can show dilatation of the aorta and pericardial effusion and tamponade, but it is associated with poor visibility in the obese patients, deformed chest, and in patients on mechanical ventilation [24]. More detailed information is obtained with transoesophageal echocardiography (TOE), especially for detection of pericardial effusion and aortic valve regurgitation, and often identification of the primary intimal tear in the aortic root. In expert hands, TOE is a powerful diagnostic tool, and its sensitivity and specificity approaching 100% [24, 25]. Coronary angiography is not indicated as a primary diagnostic tool for ATAAD. However, in many instances, ATAAD is often detected at coronary angiography, when acute coronary syndrome (ACS) is initially suspected. One need to remember that up to 30% of ATAAD patients have unknown but significant coronary artery disease that may warrant treatment and affect outcomes [26].

All patients (100%) patient came to NHIM had CT scan to diagnose ATAAD and TOE to guide the surgical treatment in operating theater. This differs from IRAD series which CT was done in almost 70% of the cases [10].

### Treatment

#### Medical treatment

The routine treatment of ATAAD in NHIM entails initial medical stabilization of pain relief and blood pressure control and followed by open surgery. It includes anti-impulsive therapy in the form of beta-blockers and vasodilators drugs to achieve systolic blood pressure of < 120 mm HG and heart rate between 60 and 80 beats per minute. This correlates with the 2014 European Society of Cardiologist (ESC) and recent 2022 American College of Cardiology/American Heart Association (ACC/AHA) guidelines on aortic diseases [27, 28]. There is a general consensus that emergency open surgical repair is the standard of care in ATAAD based on its dismal natural course, with an estimated overall case-fatality rate of 73% and in-hospital mortality rate of 58%, if not operated [27, 28].

#### Surgical treatment

Surgically, the goal of the operation is to excise the primary entry tear of the dissection, re-establish flow in the true lumen of the aorta, and repair the aortic regurgitation to avoid or reduce the lethal complications of ATAAD: rupture/tamponade, myocardial ischemia, cardiac failure related to aortic regurgitation, and life-threatening end-organ malperfusion and ischemia. This can often be achieved with a more conservative supracoronary graft, replacing the dissected ascending aorta to extensive total arch replacement (TAR) with frozen elephant trunk (FET).

All 396 (95.8%) patients received surgical management and 17 (4.1%) patients were not operated but treated medically (Table 3). Replacement of ascending aorta was the commonest operation performed in 158 (40%) patients. This was followed by root surgery in 137 (35%) of patients (Bentall: 124 (31%); valve-sparing root replacement (VSRR): 13 (3.3%)). Aortic arch surgery was performed in 101 (25.5%) of patients (partial arch replacement in 63 (16%) and TAR in 38 (9.6%)). FET was performed in 33 (8.3%) patients. The commonest concomitant procedure performed was coronary artery bypass grafting (CABG) in 86 (22%) patients. The proportion and combination of all operations are shown in Table 3. The types of operations performed for ATAAD in NHIM are pretty much similar to the IRAD series [10]. The mean cardiopulmonary bypass time was 190 min, the mean aortic cross clamping time was 125 min, and the mean hypothermia circulatory arrest time was 36 min.

**Table 3:** Surgical management of 396 operated patients with ATAAD

Primary operation	*n* (%)
Replacement of ascending aorta Aortic root procedure Bentall VSRR Hemiarch replacement Total arch replacement FET CET Combined root and hemiarch replacement Combined root and total arch replacement	158 (40%) 137 (35%) 124 (31%) 13 (3.3%) 47 (12%) 31 (8%) 26 (6.6%) 5 (1.3%) 16 (4%) 7 (2%)
Concomitant surgery	
CABG AV repair AV replacement MV repair/replacement TV repair	86 (22%) 37 (9%) 14 (3.5%) 17 (4.3%) 6 (1.5%)
Surgery time, min, mean ± SD	
CPB time, min ACC time, min Circulatory arrest time, min SACP time, min	190 ± 80 125 ± 59 36 ± 23 76 ± 64

Values are in *n* (%) and mean ± standard deviation. VSRR indicates valve sparing root replacement; *FET* frozen elephant trunk, *CET* classical elephant trunk, *AV* aortic valve, *MV* mitral valve, *TV* tricuspid valve, *CPB* cardiopulmonary bypass, *ACC* aortic cross clamp, *SACP* selective antegrade cerebral perfusion

### Early outcomes

The objective of the operation is to save the life of the patient and improve the prognosis. In contemporary reports, the overall operative early mortality from ATAAD (30-day or in-hospital) ranges from 5 to 24% [29–32] and has uniformly been around 17% in current multi-centers: GERAADA, 17%; IRAD, 18%; NORCAAD, 16% [4, 10, 33].

Overall, the early outcome of NHIM showed 71 patients (17.9%) died in the hospital, the median length of hospital stay among surviving patients was 8 days (IQR: 6–14 days), the median length of intensive care unit stay was 5 days (IQR: 3–8 days), and the median mechanical ventilation time was 24 h (IQR: 19.1–43.2 hours) (Table 4). Common complications were re-exploration for bleeding (8.0%), low cardiac output syndrome (LCOS) (6.6%), pulmonary complications (3.9%), acute kidney insufficiency (3.0 %), new-onset cerebral insufficiency (2.0%), and spinal cord injury (0.2%).

**Table 4:** Post-operative data of 396 operated patients

Early outcome	*n* (%)	
Early mortality	71 (17.9%)	
Causes		
LCOS Sepsis ARDS/respiratory failure Rupture distal aorta Acute mesenteric ischemia Acute hemorrhagic CVA Acute pancreatitis	24 (6.1%) 16 (4.0%) 11 (2.8%) 7 (1.8%) 6 (1.5%) 5 (1.3%) 2 (0.5%)	
Length of stay, median (IQR)		
Ventilation time, hour, median (IQR) ICU stay, day, median (IQR) Hospital stay, day, median (IQR)	24 (19.2–43.2) 5 (3–8) 8 (6–14)	
Complications		
Re-exploration for bleeding LCOS Pulmonary complication New-onset CVA SCI AKI/HD	33 (8.0%) 26 (6.6%) 16 (3.9%) 8 (2.0%) 1 (0.2%) 12 (3.0%)	
Predictors of early mortality	Mortality OR (95% CI)	*P* value
Hypotension Long CPB time, min Long cross-clamp time, min Additional CABG LCOS Renal failure	1.950 (1.080–3.530) 1.013 (1.009–1.017) 1.009 (1.005–1.014) 2.314 (1.347–3.974) 12.731 (1.307–124.039) 6.792 (2.343–19.683)	0.02 *P*<0.001 *P*<0.001 *P*=0.002 *P*=0.029 *P*<0.001
Late outcome		
Late mortality	35 (8.5%)	
Aortic re-intervention Re-operation Extension TEVAR	12 (3.0%) 10 (2.5%) 2 (0.5%)	

Values are in *n* (%), median (IQR) or odd ratio (*P* value). LCOS indicates low cardiac output syndrome; *ARDS* acute respiratory distress syndrome, *CVA* cerebral vascular accident, *ICU* intensive care unit, *SCI* spinal cord injury, *AKI* acute kidney injury, *HD* hemodialysis, *CI* confidence interval, *TEVAR* thoracic endovascular aortic repair

Three main causes of death were LCOS (33.8%), sepsis (22.5%), and respiratory failure (15.5%). The rest are summarized in Table 4. The significant predictors for early mortality were hypotension (*p*=0.02), long cardiopulmonary bypass time (*p*<0.001), long cross-clamp time (*p*=0.012), additional CABG (*p*=0.002), and renal failure (*p*=0.005).

### Late outcomes

**Fig. 1:**
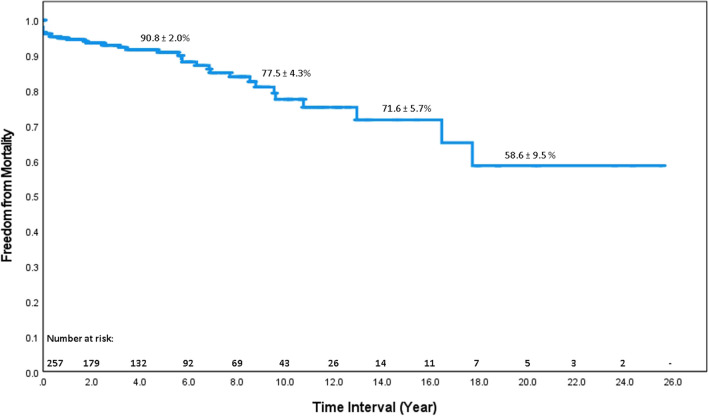
Actuarial survival of 396 operated patients. Survival at 5 and 10 years was 90.8% and 77.5% respectively

**Fig. 2:**
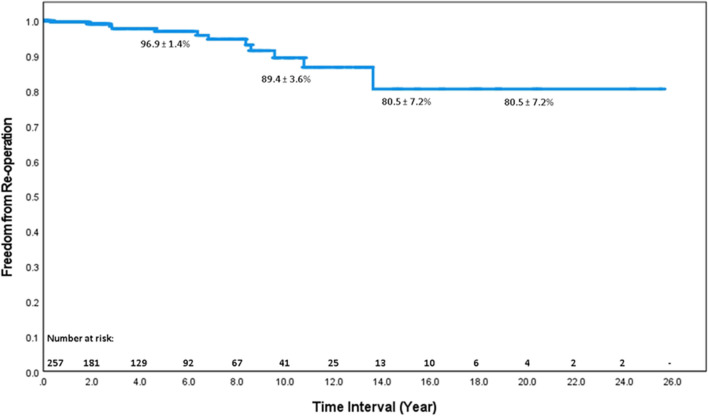
Actuarial freedom from aortic re-intervention of 396 operated patients. Freedom from re-intervention at 5 and 10 years was 96.9% and 89.4% respectively

Complete follow-up rate was 82.1%. The late death was documented in 35 patients (8.5%). Twelve cases had re-intervention (redo operation (10 cases); extension thoracic endovascular aortic repair (TEVAR) (2 cases)). Across the entire data set, survival with ATAAD treated with surgery was 90.8%, 77.5%, and 58.6% at 5.10 and 20 years post discharge respectively (Fig. 1). Freedom from aortic re-intervention was 96.9%, 89.4%, and 80.5% at 5, 10, and 20 years respectively (Fig. 2).

## Discussion

ATAAD is still considered globally as a disease with high morbidity and mortality, and remains a challenge for diagnosis and treatment. The large-scale multicentered registration study gives us a deeper understanding of the characteristics of ATAAD, and there has been a significant decrease in overall in hospital mortality in ATAAD over the past 50 years [4, 7, 10, 34–36]. Therefore, the Aortic Registry was established in accordance with the IRAD model in 2018 to improve the management of ATAAD in the NHIM and Malaysian population. We found that the population of patients with ATAAD in Malaysia undergoing surgery was younger, experienced a longer interval, and distance, from onset to arrival at the hospital but showed a relatively similar early mortality in comparison to IRAD, GERAADA, and NORCAAD series [4, 10, 34–36]. Therefore, it is necessary to establish a national multicentered database for Type A aortic dissection (TAAD) that can summarize the disease characteristics and management to improve the prognosis.

The age of patients undergoing surgery for ATAAD in Malaysia is still strikingly younger (mean age: 51 years). This study has shown that the population has a 1-decade disparity in age at onset compared with the IRAD database (mean age of 61 years). We also reviewed recent data on countries and regions with similar geographic proximity and lifestyles to Malaysia, including South Korea, Taiwan, and China. These countries have their average onset ages are lower than IRAD [10, 29, 37–39]. We just wonder whether the difference in age may be related to the economic level and the perception towards the health issues. Our results may also provide reference for some developing countries all over the world.

The presence of ATAAD with MPS is an important adverse factor for immediate and long-term survival, especially in the setting of mesenteric malperfusion [5]. We believe it is important to distinguish between ongoing arterial obstruction and arterial obstruction with ischemic end-organ dysfunction. Those patients with malperfusion but no significant adverse end-organ effects are best treated with immediate central surgical repair. Patients with established MPS should undergo fenestration with or without stenting to re-perfuse the ischemic organs with stabilization prior to open surgical repair of the ATAAD. This approach is important especially in mesenteric MPS, bilateral renal MPS, and established lower limb ischemia [5]. However, the optimal management should be individualized for each patient based on presenting features, type of malperfusion, time to surgery, and available expertise.

NHIM has almost similar spectrum of surgical treatment of ATAAD in comparison to the IRAD series [10]. Surgery performed in 95.6% of patients presented with ATAAD in NHIM series compared to 90% in IRAD series [10]. Types of operations were also comparable with the supra-coronary graft with aortic valve resuspension is the most prevalent and also the most conservative alternative treatment (NHIM (40%) vs IRAD (49%)).

When the intimal tear extends into the root, if the aortic root is aneurysmatic, or if there is uncertainty regarding the aortic valve integrity and competence of the aortic valve, the root should be replaced. This can be done as a composite graft (biological or mechanical) or as a VSRR. Patients with known or suspected connective tissue disease will probably benefit from primary aortic root replacement [30]. Distally, the surgical alternatives are numerous. A simple end-to-end anastomosis to the distal ascending aorta, with or without an aortic cross-clamp in place, is at the conservative extreme. A hemiarch replacement, including the lesser curvature of the arch, would be advocated by most; it is more radical but still comparatively comfortable and safe to perform. TAR entails reimplantation of one or more cervical vessels reimplanted as a Carrell patch or by individual grafts, or a combination. Arch replacement can be supplemented by FET, a 10–15-cm-long vascular prosthesis to direct flow preferentially into the true lumen. If the true lumen is compressed, a stent-graft (so called frozen elephant trunk) is preferable. In effect, arch replacement of any kind is certainly more challenging and time-consuming.

The management of the aortic arch in the context of ATAAD has also been under constant debate. Recent data from IRAD showed that TAR is not as widely used as hemiarch or partial arch replacement. However, many series such as GERAADA, Japanese, and Chinese Registries have shown that a more aggressive approach of aortic arch treatment can be applied without higher perioperative risk even in the onset of ATAAD [7, 29–31, 38, 39]. In our study, TAR combined with FET accounted for almost 33 cases (8.3%) which was only started in 2016. We are increasingly becoming comfortable, confident, and slowly achieving relatively low in-hospital mortality. Our indications would include pre-existing arch aneurysm/dilatation, a severely compressed true lumen inducing distal malperfusion, and a primary tear traversing the arch causing retrograde ATAAD, rendering it prone to rupture and significantly increasing the risk of cervical vessel malperfusion. In practice, arch replacement is only contemplated if deemed absolutely necessary. The FET procedure is increasingly popular approach to address complex multisegmented aortic pathologies owing to its ability to promote false lumen thrombosis and reduce the need for future operations [32].

Evolution too is seen in cannulation for cardiopulmonary bypass (CPB) and organ protection, especially coronary and cerebral. There is no solid evidence or prevailing strategy, and in fact several opposing concepts are at play regarding ideal cannulation strategies for CPB, conduct of cerebral perfusion, and proximal and distal extent of the aortic repair. Arterial cannulation for CPB must be quick, simple, safe, and versatile. For a long time, femoral artery cannulation was the primary choice in ATAAD surgery. The femoral arteries are sometimes arteriosclerotic and frequently dissected. In DeBakey type I dissections (extending beyond the aortic arch), there is a risk of malperfusion of vital organs including the brain when retrograde flow through the femoral artery, very much depending on the behavior of the intimal flap. The axillary or subclavian arteries predominantly on the right are alternative cannulation sites, are much less prone to arteriosclerosis or dissection, and provide anterograde flow in the aorta, which are also suitable to combine with antegrade cerebral perfusion during a period of hypothermic circulatory arrest (HCA) [40, 41]. However, these arteries are often less accessible and valuable time can be consumed in this pursuit. Alternatively, techniques to directly cannulate the true lumen of the dissected ascending aorta (either epi-aortic ultrasound-guided localization or transoesophageal echocardiography (TOE) of the true lumen, or by ascending aortic transection) have been proposed and they are associated with acceptable outcomes (0–15% early mortality, 4–21% stroke) [42–44]. In larger multi-center studies, no effect of cannulation sites on early mortality was observed in the GERAADA database: 15.1% vs. 18.8% for axillary vs. femoral cannulation or in the NORCAAD (19.2% vs. 18.9%) [44, 45]. Finally, less common approaches to arterial cannulation could be considered secondary alternatives, if a primary approach fails (due to arterial damage, inadequate vessel dimension, bleeding, malperfusion): innominate or carotid artery, left ventricular apex, or even the right pulmonary vein, as described recently. We in NHIM are moving towards central cannulation under the guidance of TOE with a good result.

As part of protecting the heart, we started with cold crystalloid cardioplegia but later changed to cold blood cardioplegic solution for last 15 years. It was given every 20-min interval. We are contemplating the use of Del Nido cardioplegia for aortic surgery which is still at the stage of pilot study.

Adjustment of temperature during hypothermic cardiac arrest (HCA) and brain protection is crucial. Historically, deep HCA (<18 °C) was used for cerebral and organ protection, with an attending high prevalence of neurological dysfunction and stroke, especially when the time period of HCA increased. Retrograde cerebral perfusion (RCP) by means of perfusion through a superior caval vein cannula was introduced as an adjunct to give cerebral protection [45], and favorable outcomes have been reported, from Houston: 10% stroke and 30-day mortality of 14% in 489 ATAAD repairs [45, 46]. Even so, RCP has largely been displaced by selective antegrade cerebral perfusion (SACP), delivered through direct or in direct cannulation of one, two, or three cervical vessels. Especially when combined with moderate HCA (25–30 °C), the perceived benefits include physiological (i.e., antegrade) perfusion, reduced pump-time, and reduced coagulopathy. Some series report very low prevalence of stroke and neurological dysfunction (0–6%) [47].

Conservative approaches are being increasingly challenged, and more extensive primary aortic repairs suggested, addressing associated and potential future problems of aortic dilatation (in the root, arch, proximal descending aorta, or entire thoracoabdominal aorta) that may both affect long-term survival and necessitate high-risk reinterventions. Reoperations after primary ATAAD repair are not uncommon in long-term survivors, or up to 25–30% [48, 49], and mortality is at a respectable 10–20% for elective reoperations on the proximal aorta (25–31% on the distal aorta), but as high as 67% in acute settings [48–50].

Surgical treatment of ATAAD cannot be truly evidence based. The advantages and disadvantages of each permutation of cannulation, perfusion, and surgical repair remain. Often, a conservative approach, using femoral artery cannulation, HCA with any form of cerebral perfusion, and a supracoronary graft replacing the dissected ascending aorta will suffice and provide the safest alternative. Different strategies are required to deal with the unique patient and dissection characteristics. If a risk of malperfusion through the femoral artery is envisioned or detected, an alternative arterial cannulation site should be chosen. Younger patients, or patients with connective tissue disease, may benefit most from valve sparing aortic root repair. Arch inspection during HCA may reveal a longitudinal tear motivating arch replacement, in turn an impetus for meticulous cerebral perfusion and protection, allowing ample time for repair. Thus, optimal management of a wide spectrum of ATAAD patients requires surgical adaptation and versatility, which is provided more often in dedicated aortic centers and associated with documented improved outcomes.

A successful operation needs a dedicated and structured follow-up. Pan et al. recently showed freedom from reoperation rates of 98% at 1 year and 95% at 5 years [50]. Other studies have shown freedom from reoperation of 82–94% at 5 years [50, 51]. However, Wang et al. found that patients with connective tissue disease had a 45% risk of reoperation at a median follow-up time of 2.5 years [51]. Pan et al. also found that patients with connective tissue disease had a five times higher risk of proximal reoperation [50]. This possibly indicates that a more aggressive primary approach may be feasible in patients with hereditary conditions.

## Conclusion

ATAAD is a highly deadly cardiovascular emergency. Acute surgical treatment is indicated for all patients, except for those who are moribund or severely affected by with multiorgan failures. NHIM series showed our population with ATAAD undergoing operation was younger but similar in term of spectrum of operations done, early mortality, survival, and freedom from re-intervention in comparison to IRAD, NORCAAD, and GERAADA series [4, 6, 9, 10]. We are indeed moving towards early aggressive extensive operations which includes VSRR, hemiarch replacement, and even TAR with FET to improve survival and decrease reintervention later. This includes intraoperative techniques of central or axillary canulation, moderate hypothermia, SACP, and distal open technique to improve the outcomes. Future efforts will focus on standardization of aortic registry for the entire country, and improve data collections and referral system to dedicated aortic centers. We need to improve on patient selection, perioperative management, and reduction of complications. Long-term follow-up is absolutely essential and prevention in form of identifying and treating prophylactically patients who are at particular risk of ATAAD, such as patients with aortic aneurysms due to connective tissue diseases.

## References

[1] LeMaire SA, Russel L (2011). Epidemiology of thoracic aortic dissection. Nat Rev Cardiol..

[2] Goldfinger JZ, Halperin JL, Marin ML, Stewart AS, Eagle KA, Fuster V (2014). Thoracic aortic aneurysm and dissection. J Am Coll Cardiol..

[3] Nienaber CA, Clough RE (2015). Management of acute aortic dissection. Lancet.

[4] Geirsson A, Ahlsson A, Franco-Cereceda A, Fuglsang S, Gunn J, Hansson EC (2016). The Nordic Consortium for Acute type A Aortic Dissection (NORCAAD): objectives and design. Scand Cardiovasc J..

[5] Paneer Selvam KM, Abdul SS (2022). Malperfusion in acute type A aortic dissection: how we handle the challenge?. Indian J Thorac Cardiovasc Surg..

[6] Weigang E, Conzelmann LO, Kallenbach K, Dapunt O, Karck M (2010). German registry for acute aortic dissection type A (GERAADA)–lessons learned from the registry. Thorac Cardiovasc Surg..

[7] Yamaguchi T, Nakai M, Sumita Y, Miyamoto Y, Matsuda H, Inoue Y (2020). Current status of the management and outcomes of acute aortic dissection in Japan: analyses of nationwide Japanese Registry of All Cardiac and Vascular Diseases-Diagnostic Procedure Combination data. Eur Heart J Acute Cardiovasc Care..

[8] Biancari F, Mariscalco G, Yusuff H, Tsang G, Luthra S, Onorati F (2021). European registry of type A aortic dissection (ERTAAD) - rationale, design and definition criteria. J Cardiothorac Surg..

[9] Hagan PG, Nienaber CA, Isselbacher EM, Bruckman D, Karavite DJ, Russman PL (2000). The International Registry of Acute Aortic Dissection (IRAD): new insights into an old disease. JAMA..

[10] Evangelista A, Isselbacher EM, Bossone E, Gleason TG, Eusanio MD, Sechtem U (2018). Insights from the International Registry of Acute Aortic Dissection: a 20-year experience of collaborative clinical research. Circulation..

[11] Mussa FF, Horton JD, Moridzadeh R, Nicholson J, Trimarchi S, Eagle KA (2016). Acute aortic dissection and intramural haematoma: a systematic review. JAMA.

[12] Elsayed RS, Cohen RG, Fleischman F, Bowdish ME (2017). Acute type A aortic dissection. Cardiol Clin..

[13] Nienaber CA, Eagle KA (2003). Aortic dissection: new frontiers in diagnosis and management: part I: from etiology to diagnostic strategies. Circulation.

[14] DeBakey ME, McCollum CH, Crawford ES, Morris GC, Howell J, Noon GP (1982). Dissection and dissecting aneurysms of the aorta: twenty-year follow-up of five hundred twenty-seven patients treated surgically. Surgery..

[15] Augoustides JG, Szeto WY, Desai ND, Pochettino A, Cheung AT, Savino JS (2011). Classification of acute type A dissection: focus on clinical presentation and extent. Eur J Cardiothorac Surg..

[16] Olsson C, Hillebrant CG, Liska J, Lockowandt U, Eriksson P, Cereceda AF (2011). Mortality in acute type A aortic dissection: validation of the Penn classification. Ann Thorac Surg..

[17] Pisano C, Balistreri CR, Torretta F, Capuccio P, Allegra A, Argano V (2016). Penn classification in acute aortic dissection patients. Acta Cardiol..

[18] Howard DP, Banerjee A, Fairhead JF, Perkins J, Silver LE, Rothwell PM (2013). Population-based study of incidence and outcome of acute aortic dissection and premorbid risk factor control:10- year results from the Oxford Vascular Study. Circulation..

[19] Bureau USC. International Data Base [cited 2017 25 March]. Available from: https://www.census.gov/population/international/data/idb/.

[20] Gerald TJS, Paul KLZ, Sailesh MK, John CKM. A review of aortic disease research in Malaysia. Med J Malaysia. 2019;74(1). 30846666

[21] Murty O, Tyng CCB, Kalimuthu M, Bun TS, Dany OC. Fatality due to rupture of aneurysms: autopsy review of ten years at UMMC Malaysia (1996-2005). J Forensic Med Toxicol 2007; informationGateway.php.

[22] Rogers IS, Banerji D, Siegel EL, Truong QA, Ghoshhajra BB, IrIbeck T (2011). Usefulness of comprehensive cardiothoracic computed tomography in the evaluation of acute undifferentiated chest discomfort in the emergency department (CAPTURE). Am J Cardiol..

[23] Wang GX, Hedgire SS, Le TQ, Sonis JD, Yun BJ, Lev MH (2017). MR angiography can guide ED management of suspected acute aortic dissection. Am J Emerg Med..

[24] Meredith EL, Masani ND (2009). Echocardiography in the emergency assessment of acute aortic syndromes. Eur J Echocardiogr..

[25] Shiga T, Wajima Z, Inoue T, Ogawa R (2003). Survey of observer variation in transesophageal echocardiography: comparison of anesthesiology and cardiology literature. J Cardiothorac Vasc Anesth..

[26] Tsagakis K, Konorza T, Dohle DS, Kottenburg E, Buck T, Thielmann M (2013). Hybrid operating room concept for combined diagnostics, intervention and surgery in acute type A dissection. Eur J Cardiothorac Surg.

[27] Erbel R, Aboyans V, Boileau C, Bossone E, Bartolomeo RD, Eggebrecht H (2014). ESC Guidelines on the diagnosis and treatment of aortic diseases: document covering acute and chronic aortic diseases of the thoracic and abdominal aorta of the adult. The Task Force for the Diagnosis and Treatment of Aortic Diseases of the European Society of Cardiology (ESC). Eur Heart J..

[28] Isselbacher EM, Preventza O, Black JH III, Augoustides JG, Beck AW, Bolen MA, et al. 2022 ACC/AHA Guideline for the Diagnosis and Management of Aortic Disease: A Report of the American Heart Association/American College of Cardiology Joint Committee on Clinical Practice Guidelines. Circulation. 2022;146:e334–e482. 10.1161/CIR.0000000000001106. 10.1161/CIR.0000000000001106 PMC9876736 36322642

[29] Yeh T-Y, Chen C-Y, Huang J-W, Chiu C-C, Lai W-T, Huang Y-B (2015). Epidemiology and medication utilization pattern of aortic dissection in Taiwan: a population-based study. Medicine (Baltimore)..

[30] Burgstaller JM, Held U, Mosbahi S, Stak D, Steurer J, Eckstein F (2018). A systemic review and meta-analysis: long-term results of the Bentall versus the David procedure in patients with Marfan syndrome. Eur J Cardiothorac Surg..

[31] Easo J, Weigang E, Holzl PPF, Horst M, Hoffmann I, Blettner M (2012). Influence of operative strategy for the aortic arch in DeBakey type 1 aortic dissection: analysis of the German Registry for Acute Aortic Dissection Type A. J Thorac Cardiovasc Surg..

[32] Tian DH, Ha H, Joshi Y, Yan TD (2020). Long -term outcomes of the frozen elephant trunk procedure: a systematic review. Ann Cardiothorac Surg..

[33] Czerny M, Schoenhoff F, Etz C, Englberger L, Khaladj N, Weigang E (2015). The impact of preoperative malperfusion on outcome in acute type A aortic dissection:results from the GERAADA Registry. J Am Coll Cardiol..

[34] Rylski B, Hoffmann I, Beyersdorf F, Suedkamp M, Siepe M, Nitsch B (2014). Acute aortic dissection type A: age-related management and outcomes reported in the German Registry for Acute Aortic Dissection Type A (GERAADA) of over 2000 patients. Ann Surg..

[35] Trimarchi S, Eagle KA, Nienaber CA, Rampoldi V, Jonker FHW, Vincentiis CD (2010). Role of age in acute type A aortic dissection outcome: Report from the International Registry of Acute Aortic Dissection (IRAD). J Thorac Cardiovasc Surg..

[36] Boening A, Karck M, Conzelmann LO, Easo J, Kruger T, Rylski B (2017). German Registry for Acute Aortic Dissection Type A: structure, results, and future perspectives. Thorac Cardiovasc Surg..

[37] Zhu Y, Lingala B, Baiocchi M, Tao JJ, Arana VT, Khoo JW (2020). Type A aortic dissection-experience over 5 decades: JACC Historical Breakthroughs in Perspective. J Am Coll Cardiol..

[38] Zhao R, Qiu J, Dai L, Song J, Fan S, Cao F (2022). Current surgical management of acute type A aortic dissection in China. JACC Asia J Am Coll Cardiol..

[39] Ahn J-M, Kim H, Kwon O, Om SY, Heo R, Lee S (2019). Differential clinical features and long-term prognosis of acute aortic syndrome according to disease entity. Eur Heart J..

[40] Strauch JT, Spielvogel D, Lauten A, Lansman SL, Mcmurtry K, Bodian CA (2004). Axillary artery cannulation: routine use in ascending aorta and aortic arch replacement. Ann Thorac Surg..

[41] Sabik JF, Nemeh H, Lytle BW, Blackstone EH, Gillinov AM, Rajeswaran J (2004). Cannulation of the axillary artery with a side graft reduces morbidity. Ann Thorac Surg..

[42] Conzelmann LO, Kayhan N, Mehlhorn U, Weigang E, Dahm M, Vahl CF (2009). Reevaluation of direct true lumen cannulation in surgery for acute type A aortic dissection. Ann Thorac Surg..

[43] Khaladj N, Shrestha M, Peterss S, Strueber M, Karck M, Pichlmaier M, et al. Ascending aortic cannulation in acute aortic dissection type A: the Hannover experience. Eur J Cardiothorac Surg. 2008;34:792–6. 10.1016/j.ejcts.2008.05.014 18579405

[44] Tiwari KK, Murzi M, Bevilacqua S, Glauber M (2010). Which cannulation (ascending aortic cannulation or peripheral arterial cannulation) is better for acute type A aortic dissection surgery?. Interact Cardiovasc Thorac Surg..

[45] Ueda Y, Miki S, Kusuhara K, Okita Y, Tahata T, Yamanaka K (1990). Surgical treatment of aneurysm or dissection involving the ascending aorta and aortic arch, utilizing circulatory arrest and retrograde cerebral perfusion. J Cardiovasc Surg (Torino)..

[46] Tian DH, Weller J, Hasmat S, Oo A, Forrest P, Kiat H (2018). Adjunct retrograde cerebral perfusion provides superior outcomes compared with hypothermic circulatory arrest alone: a meta-analysis. J Thorac Cardiovasc Surg..

[47] Panos A, Murith N, Bednarkiewicz M, Khatchatourv G (2006). Axillary cerebral perfusion for arch surgery in acute type A dissection under moderate hypothermia. Eur J Cardiothorac Surg..

[48] Bachet JE, Termignon JL, Dreyfus G, Goudot B, Martinelli L, Piquois A (1994). Aortic dissection. Prevalence, cause, and results of late reoperations. J Thorac Cardiovasc Surg..

[49] Dell’Aquila AM, Pollari F, Fattouch K, Santarpino G, Hillebrand J, Schneider S (2017). Early outcomes in re-do operation after acute type A aortic dissection: results from the multicenter REAAD database. Heart Vessels.

[50] Pan E, Gudbjartsson T, Ahlsson A, Fuglsang S, Geirsson A, Hansson EC (2018). Low rate of reoperations after acute type A aortic dissection repair from The Nordic Consortium Registry. J Thorac Cardiovasc Surg..

[51] Wang H, Wagner M, Benrashid E, Keenan J, Wang A, Ranney D (2017). Outcomes of reoperation after acute type A aortic dissection: implications for index repair strategy. J Am Heart Assoc..

